# Supplementary material for: Tumor-Infiltrating Immune Cells and HLA Expression as Potential Biomarkers Predicting Response to PD-1 Inhibitor Therapy in Stage IV Melanoma Patients
Source: Biomolecules. 2024 Dec 16;14(12):1609. doi: 10.3390/biom14121609 (PMC11674713; doi:10.3390/biom14121609)
Supplement: Supplementary file 1 [file biomolecules-14-01609-s001.zip › biomolecules-3326921-supplementary.pdf]

Table S1. Clinicopathological data of individual patients and heatmap of immunohistochemistry results

Patient no.	ECOG	Line	BRAF	LDH	Anti-PD-1 drug	BORR	PFS(mo)	Progr	OS(mo)	Death	HC10	HCA2	B2M	EMR	HLAII	CD8	CD45RO	CD20	FOXP3	CD134	CD137	CD103	NKp46	PD-1	PD-L1
Resp1	0	4	2	0	Pembrolizumab	CR	16	1	68	0	1,0	1,0	1,0	1,5	11	65,6	134,4	6,4	14,4	1,8	4,2	86,4	3,5	60,8	11,2
Resp2	0	1	1	0	Nivolumab	CR	89	0	90	0	2,0	2,0	2,0	2,0	87	33,6	724,8	22,4	33,6	6,7	2,7	36,8	4,2	20,8	36,8
Resp3	0	1	1	0	Pembrolizumab	CR	47	1	91	0	2,0	1,3	1,0	2,0	3	123,2	752,0	195,2	100,8	15,8	25,8	102,4	9,0	80,0	75,2
Resp4	0	1	2	0	Pembrolizumab	CR	6	1	21	1	2,0	1,7	2,0	2,0	0	84,8	507,2	38,4	81,6	68,0	8,6	225,6	1,1	126,4	128,0
Resp5	0	3	1	0	Nivolumab	CR	47	1	87	0	0,7	1,3	0,5	1,5	0	56,0	107,2	8,0	17,6	5,3	2,6	41,6	2,1	11,2	19,2
Resp6	0	1	1	0	Pembrolizumab	CR	39	1	45	1	1,8	1,7	1,7	1,8	33	204,8	521,6	30,4	44,8	24,0	5,3	56,0	1,3	190,4	161,6
Resp7	0	1	2	0	Pembrolizumab	CR	21	1	75	1	0,5	1,0	1,0	1,0	4	54,4	110,4	0,0	9,6	0,0	4,0	38,4	0,0	27,2	14,4
Resp8	0	1	1	0	Pembrolizumab	CR	62	0	63	0	2,0	2,0	2,0	2,0	4	206,4	769,6	17,6	17,6	5,9	36,6	206,4	14,4	393,6	705,6
Resp9	0	1	1	0	Pembrolizumab	CR	39	0	40	0	1,9	2,0	1,9	1,9	3	84,8	225,6	17,6	20,8	4,5	10,7	115,2	3,7	44,8	67,2
Resp10	0	1	1	0	Pembrolizumab	CR	25	1	39	1	1,0	0,3	1,1	1,1	0	147,2	425,6	54,4	41,6	5,8	15,5	73,6	9,4	96,0	268,8
Resp11	0	2	2	1	Pembrolizumab	CR	58	0	60	0	2,0	1,8	2,0	2,0	0	12,8	110,4	11,2	4,8	1,3	1,1	17,6	0,2	4,8	1,6
Resp12	0	1	1	0	Pembrolizumab	CR	15	1	15	1	1,0		2,0	2,0	3	208,0	408,0	27,2	20,8	0,8	6,4	190,4	5,3	134,4	104,0
Resp13	0	1	1	0	Pembrolizumab	CR	22	0	22	0	2,0	2,0	2,0	2,0	54	540,8	681,6	40,0	48,0	1,9	9,0	243,2	7,2	329,6	211,2
Resp14	0	1	1	0	Nivolumab	CR	29	1	43	0	0,2	0,3	0,0	0,4	0	193,6	257,6	41,6	43,2	9,3	8,3	38,4	1,9	72,0	315,2
Resp15	0	2	1	0	Nivolumab	PR	17	1	24	1	2,0	2,0	2,0	2,0	0	32,0	683,2	11,2	40,0	2,6	11,4	20,8	5,6	6,4	144,0
Resp16	1	1	1	1	Nivolumab	PR	90	0	90	0	1,0	1,0	1,0	1,0	14	24,0	64,0	19,2	14,4	6,7	10,7	24,0	0,8	20,8	19,2
Resp17	0	2	2	1	Nivolumab	PR	79	0	79	0	0,7	1,6	0,9	1,4	3	20,8	84,8	3,2	6,4	2,4	1,4	24,0	3,2	32,0	9,6
Resp18	0	1	1	0	Nivolumab	PR	16	1	49	1	1,5	1,5	1,7	2,0	0	150,4	627,2	56,0	52,8	1,9	13,3	246,4	4,0	222,4	97,6
Resp19	0	2	2	0	Pembrolizumab	PR	66	0	68	0	0,6	1,0	1,0	1,2	32	310,4	400,0	12,8	12,8	4,3	3,2	208,0	1,6	120,0	57,6
Resp20	0	1	1	0	Pembrolizumab	PR	35	1	66	1	0,0	0,7	1,0	1,0	0	40,0	92,8	4,8	9,6	1,6	1,1	19,2	0,3	17,6	1,6
Resp21	0	2	1	0	Pembrolizumab	PR	4	1	13	1	1,0	0,5	2,0	2,0	0	28,8	76,8	4,8	28,8	18,7	2,4	17,6	0,0	19,2	9,6
Resp22	0	1	1	1	Pembrolizumab	PR	12	1	15	1	0,9	2,0	1,5	1,9	0	70,4	441,6	30,4	20,8	10,6	4,2	30,4	0,5	38,4	105,6
Resp23	0	1	1	0	Nivolumab	PR	9	1	38	1	2,0	2,0	2,0	2,0	4	107,2	678,4	19,2	24,0	6,6	11,4	80,0	1,0	97,6	176,0
Resp24	0	1	1	0	Nivolumab	PR	20	1	47	1	0,0	0,5	0,5	0,0	0	48,0	344,0	17,6	16,0	3,5	11,2	64,0	1,0	70,4	177,6
Resp25	0	2	2	0	Pembrolizumab	PR	48	1	68	1	1,2	2,0	1,8	1,8	33	180,8	443,2	6,4	54,4	7,7	4,2	169,6	3,7	59,2	75,2
Resp26	0	1	1	0	Pembrolizumab	PR	6	1	34	0	2,0	2,0	2,0	2,0	4	129,6	299,2	12,8	24,0	3,0	3,5	84,8	2,7	96,0	62,4
Resp27	0	1	1	0	Pembrolizumab	PR	31	0	33	0	1,2	0,7	1,2	1,0	23	321,6	430,4	64,0	32,0	4,3	12,5	212,8	12,6	257,6	123,2
Resp28	0	1	2	1	Pembrolizumab	PR	7	1	21	1	1,0	1,0	1,5	1,5	24	374,4	616,0	123,2	44,8	7,7	37,3	78,4	16,3	152,0	137,6
Nonresp1	1	1	1	1	Nivolumab	SD	5	1	9	1	1,0	0,5	0,0	1,0	0	48,0	584,0	17,6	36,8	10,7	15,5	73,6	2,9	17,6	52,8
Nonresp2	0	2	2	1	Pembrolizumab	SD	11	1	51	1	0,5	1,1	2,0	1,2	0	48,0	86,4	1,6	17,6	1,4	1,8	35,2	0,0	38,4	11,2
Nonresp3	0	1	1	1	Pembrolizumab	SD	3	1	62	0	0,0	1,0	0,5	0,5	0	33,6	387,2	0,0	6,4	2,9	2,6	20,8	0,0	16,0	25,6
Nonresp4	1	1	1	1	Pembrolizumab	SD	3	1	46	0	0,0	1,2	1,2	1,0	0	28,8	158,4	12,8	19,2	3,2	1,8	9,6	1,6	12,8	27,2
Nonresp5	1	5	1	1	Nivolumab	PD	3	1	7	1	0,1	0,3	0,0	0,4	0	25,6	83,2	6,4	11,2	4,0	6,6	35,2	6,6	14,4	12,8
Nonresp6	0	2	2	1	Nivolumab	PD	2	1	4	1	2,0	2,0	1,6	2,0	0	11,2	137,6	1,6	6,4	1,8	4,8	1,6	6,6	6,4	43,2
Nonresp7	0	1	1	0	Nivolumab	PD	3	1	10	1	0,5	1,5	1,0	1,0	0	1,6	206,4	1,6	16,0	0,0	4,2	11,2	0,5	1,6	0,0
Nonresp8	0	1	1	1	Nivolumab	PD	2	1	5	1	0,0	0,0	0,0	0,0	0	14,4	120,0	1,6	12,8	2,2	3,5	8,0	3,2	3,2	4,8
Nonresp9	0	2	1	0	Nivolumab	PD	1	1	2	1	1,0	2,0	1,0	2,0	0	131,2	252,8	8,0	41,6	0,8	17,0	60,8	1,0	46,4	20,8
Nonresp10	1	1	1	0	Nivolumab	PD	3	1	15	1	2,0	2,0	2,0	2,0	97	347,2	774,4	27,2	108,8	6,4	16,0	131,2	10,6	326,4	123,2
Nonresp11	0	1	1	1	Pembrolizumab	PD	3	1	4	1	2,0	1,7	2,0	2,0	12	14,4	182,4	11,2	8,0	1,1	0,2	46,4	1,0	4,8	1,6
Nonresp12	0	1	1	0	Nivolumab	PD	3	1	11	1	1,2	1,2	1,5	1,1	1	68,8	262,4	27,2	16,0	2,7	1,8	24,0	0,5	0,0	6,4

Red/green color of the heatmap label high/low expression or high/low immune cell density.

Resp: responder, Nonresp: nonresponder, ECOG: Eastern Cooperative Oncology Group, LDH: lactate dehydrogenase (0 vs. 1: lower vs. higher than the upper limit of normal), BORR: best overall response, PFS(mo): progression-free survival (months), Progr: progression, OS(mo): overall survival(months)
